# A Mixed-Methods Outcomes Evaluation Protocol for a Co-Produced Psychoeducation Workshop Series on Recovery from Psychosis

**DOI:** 10.3390/ijerph192315464

**Published:** 2022-11-22

**Authors:** Ying Ying Lee, Wei Ler Koo, Yi Fong Tan, Vanessa Seet, Mythily Subramaniam, Suying Ang, Charmaine Tang

**Affiliations:** 1 Research Division, Institute of Mental Health, Singapore; 2 Saw Swee Hock School of Public Health, National University of Singapore, Singapore; 3 Early Psychosis Intervention Programme, Institute of Mental Health, Singapore

**Keywords:** co-production, recovery college, outcome evaluation, implementation, mental health, psychosis

## Abstract

Co-production in mental health is a relatively new approach to designing and delivering mental health services, which involves collaboration amongst professionals, persons in recovery, and their caregivers to provide services. The aim of this protocol paper is to detail the implementation and evaluation of a co-produced workshop series named *Broken Crayons*. Collaborating with an early intervention program for first-episode psychosis, the study team and peer volunteers generated a co-production framework based on their experience of co-producing 11 workshops. This paper also outlines a protocol to evaluate *Broken Crayons*, a psychoeducation workshop series co-created and co-delivered by mental health professionals, persons in recovery, and their caregivers. Indicators on personal recovery, mental wellbeing, community integration, etc., are included as outcomes. Two-tailed, paired *t*-tests will be used to compare pre- and post-workshop survey data. Focus group discussions will also be conducted to gather subjective experiences of participants of the *Broken Crayons* workshops. Cost-savings of co-production by Recovery Colleges are discussed. The implications of using co-production to foster citizenry in persons living with first-episode psychosis are discussed in the context of social causation and social drift theories. Taken together, we argued that co-production is not just a passing trend, but a moral imperative for inclusive and equitable mental health service design and delivery.

## 1. Introduction

Co-production in mental healthcare is one of the latest approaches to the design and delivery of mental health services [1]. The term “co-production” was first coined by Nobel laureate Elinor Ostrom and colleagues, to solve youth social justice issues in the Chicago area during the 1970s [2]. The ideal was later operationalized by law professor, Edgar Cahn, which has evolved into a key working principle in the UK’s mental healthcare system today [3]. A working definition of co-production in mental health is “delivering public services in an equal and reciprocal relationship between professionals, people using services, their families and their neighbors” [4]. A key feature in co-production is the building of reciprocal relationships amongst mental health professionals, the beneficiaries of services, and other members in the community via partnerships. While some approaches to mental healthcare may focus on efficiency and cost-saving, co-production focuses on relationships, power-sharing, and knowledge-sharing between professionals and the lay-stakeholders [5].

**Figure 1:**
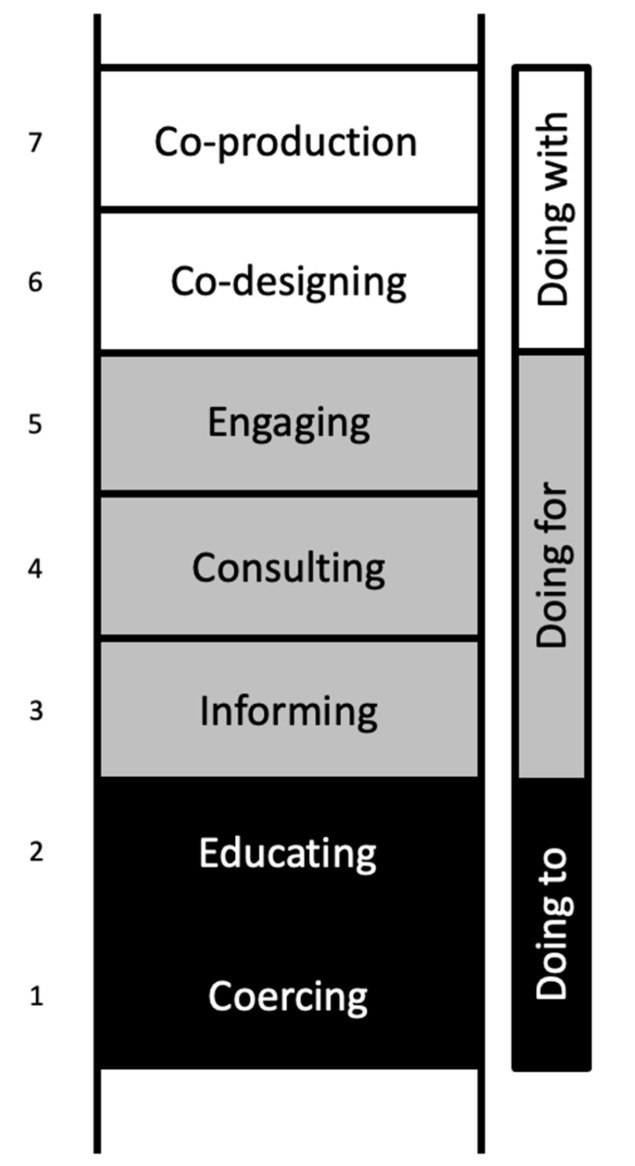
A modified Ladder of Participation to depict the levels of patient participation on mental health service design and delivery. The original Ladder of Participation was first described by Sherry Arnstein in context of citizen participation in 1969 [6]. Building upon Arnstein’s ladder, the new economics forum (nef) adapted the ladder into the context of mental health service delivery in the UK [7].

Co-production can be portrayed by a modified Ladder of Participation (Figure 1). The Ladder of Participation was first mooted by Sherry Arnstein [6] to illustrate different levels of public participation and social activation. This ladder was adapted by the New Economics Foundation (NEF) to depict co-production. NEF identified the first two rungs of the ladder as “coercing” and “educating”, where services were “done to” recipients in a strict unidirectional level from professional to beneficiaries. Moving up the rungs to levels three to five lie “informing”, “consulting”, and “engaging”, where services were “done for” recipients. There may be some level of input from the recipients in terms of the services provided, but final decisions were still made by professionals. In the top rungs of the ladder, levels six and seven, lie “co-designing” and “co-production”, where professionals and care recipients come together to design and deliver services together [7]. Services were “done with” its recipients shoulder to shoulder. In Arnstein’s original ladder, there was a level 8, where she advocated for citizen control, in which communities are managed and administered by the people, for the people [6]. While this ideal seems to be reasonable in the context of citizenry advocacy work, such a model might not be suitable for mental healthcare. The involvement of multi-stakeholders to co-design and co-produce mental healthcare services remains the most viable and logical route. Co-production is not a means to abolish the existing mental healthcare system; it instead aims to enhance the current system into one that is more humane and relevant [8]. For instance, co-production could include the voices of persons in recovery and their caregivers into the care model, so that the services stay relevant, relatable, and humanistic.

Within mental healthcare, there exists a power differential between professionals providing care and beneficiaries receiving care [8]. This power differential is especially jarring within mental health because of the nature of mental health conditions and the social and internalized stigma that comes with being afflicted with a mental health condition. In addition to the medical aspects, living with a diagnosis of mental health conditions has psychological and social consequences. Various scholars have described a phenomenon known as “social drift”, where a person living with chronic conditions, including mental health conditions, moves down the social ladder due to reduced social desirability [9,10,11,12]. Reports of discrimination against persons with mental health conditions in social and occupational contexts have been reported worldwide [13,14]. As such, co-production is not simply a trendy approach to mental healthcare but a moral imperative to involve patients in their own care [8]. Returning agency and autonomy to patients in their own care is of paramount importance.

Having said that, it does not mean that mental healthcare is obsolete. In fact, mental health professionals play important roles in creating the environment of empowerment and hope for their beneficiaries, especially at the initial stages of being diagnosed with a mental health condition. This is where co-production comes in: to develop mental health services that are inclusive and equitable with those afflicted by mental health conditions. It is crucial to always involve people who are recovering from mental health conditions to co-design and co-produce mental health services, because without their voice, it is easy to lose touch with the realities of recovery, particularly in settings such as tertiary psychiatric hospitals. This is because mental health professionals come into contact with patients at their worst day in and day out. When the patients recover, they are discharged to community care. Due to this lack of interaction with patients who have maintained recovery, professionals working in tertiary mental healthcare may have a negatively skewed view of their patients [15]. Barriers to personal recovery in mental healthcare include systems without trauma sensitivity, a lack of advocacy support, and limited access to psychosocial approaches [16]. As a result, many professionals in the tertiary psychiatric settings lose sight of the full clinical picture, which included recovery from serious mental illness. Co-production is a way in which professional expertise and lived experience are melded together; theories on recovery could be contextualized by persons in recovery’s lived experience.

The Recovery College, pioneered in the UK, is one way to operationalize co-production in mental healthcare [17]. Recovery Colleges were first developed by teams in London and Sussex [18]. A decade after the idea of Recovery Colleges was first mooted, there are now more than 75 Recovery Colleges established all over the world [18]. Outcomes evaluation on co-produced recovery workshops from the UK suggest that they improve patient-reported outcomes such as personal recovery, mental wellbeing, and quality of life [19,20]. Co-production is easy to conceptualize but difficult to operationalize. While there are many white papers and guides that expound on co-production [5], locating a useful guide on the nuts and bolts of co-production is far more difficult. Another challenge is that co-production is a relatively new approach to mental healthcare in Singapore. Finding a culturally relevant and sensitive guide to the Singaporean context proved to be difficult. As such, a team, made up of staff and volunteers, at the Institute of Mental Health (IMH) came together to develop a framework suited to the specific context of the Early Psychosis Intervention Programme (EPIP) in the IMH to co-design and co-produce psychoeducation workshops on recovery from psychotic disorders. More details about this framework are covered in the Section 2.

There are two arms in this study: an implementation arm, where mental healthcare professionals, patients, and their caregivers come together to design and deliver psychoeducation workshops, and an evaluation arm, where these workshops are then evaluated for their effectiveness. In sum, the three aims are (1) to unite mental health professionals, patients, and their caregivers in the design and delivery of psychoeducation workshops pertaining to recovery from psychotic disorders; (2) to evaluate the effectiveness of these workshops at improving participants’ personal recovery, mental wellbeing, sense of social inclusion, and social stigma distance against persons with mental health conditions; finally, (3) to examine participants’ perspectives and experiences after attending the co-produced workshops. These psychoeducation workshops are referred to as *Broken Crayons* in this manuscript.

## 2. Materials and Methods

### 2.1. Context of Implementation and Evaluation

Located in Singapore, the Institute of Mental Health (IMH) is a 2000-bed tertiary psychiatric hospital. Serving a population of 5.5 million Singapore residents [21], the IMH is the only tertiary psychiatric hospital in Singapore. The IMH serves a diverse multi-lingual, multi-cultural population made up of Chinese, Malays, Indians, Eurasians, and persons of other ethnicities, providing care for more than 50,000 outpatients per year and more than 1700 inpatients per day. Nested within the IMH is EPIP, a multi-disciplinary, early intervention team made up of psychiatrists, case managers, occupational therapists, peer support specialists, and other allied health workers to support young people aged between 12 and 40 years with first-episode psychosis. In addition to medical care, EPIP provides a comprehensive psychosocial program to help young persons in recovery from early psychosis and integrate them back into their age-appropriate roles, at school or work. EPIP has a host of psychosocial programs that help young persons in recovery from first-episode psychosis regain psychosocial functioning. Psychosocial workshops pertaining to the journey of recovery are co-created and co-produced with mental health professionals, persons in recovery, and their caregivers. *Broken Crayons* is marketed by word of mouth, through the EPIP, community partners, and through the events page of the IMH’s website. Anyone with an interest to learn about psychosis is invited to participate in the workshop series.

### 2.2. Implementation: Workshop Contents

The *Broken Crayons Still Color* workshops *(Broken Crayons)* are targeted at clients of the EPIP and their caregivers. However, these workshops are also open to members of the public with an interest to learn more about psychotic disorders. The rationale behind this is to promote social and community integration for young persons in recovery from psychosis, and for members of the public to interact with persons in recovery to debunk stereotypes and misconceptions about people living with psychotic disorders. *Broken Crayons* was modeled after the co-production approach of Recovery Colleges in the UK [17]. In the UK, Recovery Colleges provide a whole suite of recovery education workshops for mental health professionals, caregivers, and persons in recovery, covering content from five basic domains: understanding mental health issues and treatment options, rebuilding life with mental health challenges, developing life skills, capacity building among the peer workforce, and family and friends [17]. *Broken Crayons* could be considered as analogous to the courses on understanding mental health issues and treatment options and rebuilding of life with mental health challenges in Recovery Colleges.

### 2.3. Implementation: Broken Crayons Synopsis

*Broken Crayons* was first developed in early 2019 as an in-person workshop series by a group of like-minded volunteers from a ground-up initiative, SOAR (School of Ability and Recovery) [22]. The inaugural run of *Broken Crayons* was from July 2019 to December 2019. Due to the COVID-19 pandemic, *Broken Crayons* was repurposed into an online/hybrid workshop series for this outcome evaluation study. The study team members have planned three runs of *Broken Crayons* (March to April 2022, July to August 2022, and October to November 2022) to meet the recruitment target for the evaluation study. Online sessions will be held via the Zoom videoconferencing platform, while in-person sessions will be held at community sites accessible to most attendees. This six-part workshop series covers the bio-psycho-social aspects of recovery from psychotic disorders. Table 1 shows a summary of the six workshops.

The workshop starts with the topic “Understanding Psychosis”, where a psychiatrist will give a 15 to 30-min psychoeducation talk to the participants, followed by a panel discussion comprising a person in recovery, caregiver, case manager, and psychiatrist. The series continues into “Journeying with Psychosis 1 & 2”, where participants will experience a pre-recorded interactive play that follows a protagonist, “Jamie”, through her first encounter with auditory hallucinations and delusions. In these two sessions, participants will first view the interactive play; facilitators will then lead and guide the group to process Jamie’s experiences and relate it back the journey of recovery from psychosis. Thereafter, participants will be taken through three main aspects of recovery from psychosis: medications, mindset, and mind. In this session, participants will get to go more in-depth into managing medications, keeping a positive mindset during recovery, and managing challenging thoughts and emotions that come with living with a diagnosis of psychotic disorders. The workshop “Disclosure of Medical History” will involve a panel discussion made up of a person in recovery, an employer, a caregiver, and an allied health professional. Together, the panelists will be having an honest conversation around the disclosure of mental health conditions in the workplace. The final session of the workshop series “Family & Friends” is about bringing together family and friends of persons in recovery to create a collaborative artwork and share stories about how the workshop series has made a difference to the workshop participants.

### 2.4. Implementation: A Framework for Co-Producing Psychoeducation Workshops in EPIP

The idea to co-create a co-production framework came up during a volunteer meeting session, where we were struggling to include diversity in our co-production sessions—the group of peer volunteers were unclear about how mental health professionals could be involved in the co-production process. Hence, we took a step back to come up with a framework to operationalize co-production of psychoeducation workshops in the context of EPIP. This framework was developed based on the development of 11 co-produced workshops. Over four months (August to November 2021), the team had seven sessions with more than 10 staff and volunteers to co-create the following co-production framework. Online tools such as Google Jamboard and Miro were used to facilitate the generation of ideas. An initial scaffold was generated with reference to publications by the International Association for Public Participation (IAP2) [23] and unpublished participatory frameworks to structure the ideas. The work that we do was informed by a guidance manual developed by Jones [24], where she had developed 7 self-assessment questions that we have applied in our work to support meaningful involvement of peers. While we did not explicitly state strategies to avoid or prevent tokenistic involvement by participants, the entire framework was developed to guide meaningful involvement of persons in recovery, caregivers, and mental health professionals in the development of the co-produced workshops. In the manual by Jones, she had developed 7 self-assessment questions that we have applied in our work to involve peers (Table 2).

**Figure 2:**
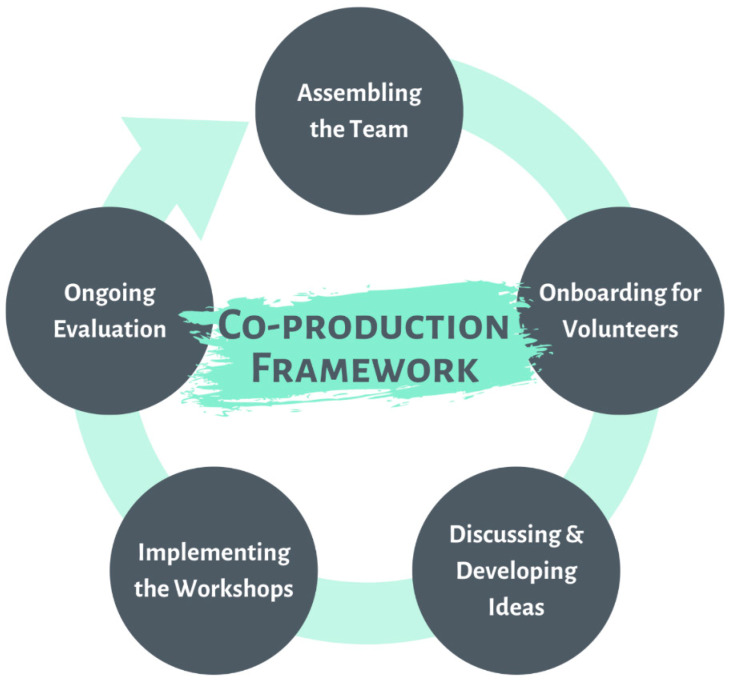
A framework for co-production to co-create and co-develop psychoeducation workshops for people with an interest in psychotic disorders. Five steps are identified in this framework (1) Assembling the Team, (2) On-boarding of Volunteers, (3) Discussing and Developing Concepts, (4) Implementation of the Workshops, and (5) Ongoing Evaluation.

Figure 2 summarizes the co-production framework into a cyclical model, while Supplementary File S1 contains the key points of the co-production framework.

### 2.5. Assembling the Team

The framework begins with *Assembling the Team*, which details who, where, and how to source for team members and areas of consideration when planning for co-production. Team members are usually sourced from two places: previous workshop participants and members of the EPIP team. At the end of every workshop run, a call for volunteers would be made to invite participants to join as volunteers. This group made up the core group of volunteers. They usually consist of peers (~50% of group in numbers), caregivers (~25%), and members of the public with an interest in mental health (~10%). Another source of volunteers come from the multidisciplinary team of EPIP (~15%). Peer support specialists, psychiatrists, case managers, and occupational therapists were invited to join on an ad hoc basis, lending their professional expertise to various workshops. For instance, venue matters. Convenience, accessibility, and finding a neutral ground are deemed as important for our volunteers. The corresponding levels of involvement highlighted that perspective matters: what may be convenient, accessible, and neutral from a mental health professional’s perspective may not be the same from a patient’s. In the highest level of involvement, the co-production framework aims to fulfil elements of convenience, accessibility, and neutrality from (almost) everyone’s perspective. Teams are made up of people, hence being intentional that the invitation, power sharing, and diversity in the group matter. In the highest level of involvement, this framework aims to invite people from various ethnic, cultural, and age groups and stages of recovery into the co-production group. Flexibility in team organization in terms of group formation and members taking turns to lead is taken into consideration. The volunteer group is run on a very flat hierarchy, meaning that we relate to every volunteer as a human being first before they are peers, caregivers, professionals, or others. For example, when breaking the ice for volunteers, we avoid getting members to introduce their “roles” in context of the EPIP program. Instead, we usually set prompters such as “What is one interesting fact about yourself that few people know?” as the icebreaker game. At the heart of co-production is building meaningful relationships. To run co-production in an equitable manner, everyone needs to set aside their existing differences in power dynamics (patient versus professional; caregivers versus members of public) to co-produce and co-create.

### 2.6. On-Boarding of Volunteers (i.e., Team Members Assembled to Form a Team)

Proper on-boarding of volunteers is crucial for the success of the co-production effort. Five main areas are identified in this step: (1) Volunteer induction, (2) Volunteer roles, (3) Stage-by-stage involvement, (4) Development and retention, and (5) Commitment level, Table 3.

At the highest involvement level, each new volunteer is tagged with a more experienced volunteer to learn the ropes, and people are welcomed at every stage of recovery, just as they are. A strengths-based approach is taken to identify roles for the volunteers, matching specific roles to the specific strengths of each volunteer. Involvement for volunteers can take place in a staged process; beginner volunteers can take on smaller roles until they are ready to lead sessions and be the main facilitator. Long-term development and training plans for volunteers are ongoing based on training needs assessments. Commitment levels of volunteers can be flexible and based on their availability and current stage of recovery/life.

### 2.7. Discussing and Developing Concepts

Five themes are identified in this step. Mode of communication, leadership in groups, conflict resolution, content development, and process development are discussed. Collaborative tools such as Miro and Google Docs are used for every discussion, which gives every volunteer a means to reach everyone and contribute to the development and discussion of workshop ideas. Volunteers, consisting of peers, caregivers, and professionals, and others, are involved to draw from their lived experiences and professional expertise to develop the content. The decision-making process in the development of these workshops are shared by members in the group; in every co-production session, the group comes to a consensus by discussion and shared decision-making. The contents of workshops are re-evaluated constantly by workshop facilitators to keep them fresh and relevant to the times. Lived experience of the workshop facilitators is actively integrated into workshop content. A balance between content and process development is struck to ensure participants acquire new knowledge, but also connect what they learn with their emotions.

### 2.8. Implementation of Workshops

Three areas are identified as essential in this workshop implementation step—delivery method, group dynamics, and group culture. At the highest involvement level, there are more hands-on discussions and activities to collaboratively generate ideas and share knowledge and lived experiences between participants and facilitators. There should be dynamic interactions between facilitators and participants, which creates space for participants to initiate the lead in discussions. Balancing positivity and negativity in a nuanced and mature way is also important in the development of the group culture: members should be able to come as they are and discuss problems in a way that is edifying to themselves and others. Narratives of all kinds are welcomed: there are no “model answers” to the kind of story that our participants can share.

### 2.9. Ongoing Evaluation

Of the five steps, ongoing evaluation is the trickiest and one that is mostly a work in progress. Even though it is presented as the “final” step of the framework, it is present at almost all steps in the background. Qualitative and quantitative feedback is constantly obtained from volunteers and participants to understand the process of implementation (process evaluation) to identify opportunities for growth and improvement. Outcome evaluation planning needs to be integrated right from the beginning of co-production to identify specific outcomes to evaluate. Capacity building is a key area to involve non-research trained volunteers to contribute to the process of evaluation. Communication of evaluation results can be regular and creative. Beyond the one-way communication of results, volunteers may be involved in co-authoring research communication pieces, such as abstracts, presentation, and academic manuscripts, to achieve higher levels of involvement and ownership of the project.

### 2.10. Evaluation: A Mixed-Methods Outcomes Evaluation Design

#### 2.10.1. Participants

Participants of the *Broken Crayon* workshop series will be invited to join the evaluation study. However, the participants will be separated into two demographics: patient and non-patient. The patient participants will be recruited from the IMH’s EPIP. Potential participants will be first contacted by their attending healthcare professional before follow-ups from the study team’s members if they are agreeable for the study. Inclusion criteria for patient participants include participants aged 16 to 40 inclusive, who are diagnosed with any psychotic disorders and are currently receiving treatment from EPIP. Additionally, participants must be English-speaking, and parental consent must be obtained for all participants between the age of 16 and 20 inclusive.

After workshop registration, non-patients will first be contacted by a research executive working closely with both EPIP and research teams to ask for their interest to join the research study during registration for the workshop. The research study team will then follow up with the non-patients for recruitment into the study. Inclusion criteria for non-patient participants include English-speaking participants of age 21 years and above, who are not currently seeking any treatment from EPIP and are not staff belonging to the IMH. We had included participants above 16 years and below 21 years in the patient participants because we found that there were a significant proportion of patients with first-episode psychosis between the ages of 16 and 20 years in the pilot run. However, for non-patient participants, it is usually the adult caregivers who sign up for the pilot run. Hence, we had a different criterion for age for inclusion in the evaluation study. Workshop participants who decline participation in this study, or who do not meet the inclusion criteria will be excluded from the study if they do not meet any one of the inclusion criteria.

Different scales will be administered to the patient and non-patient participants. Patient participants will furnish data on personal recovery (Questionnaire about the Process of Recovery), mental wellbeing (Short Warwick–Edinburgh Mental Well-Being Scale), and community integration (Community Integration Measure); the Social Distancing Scale will be used to measure respondents’ willingness to be socially close to persons with mental health conditions. Clinical assessments such as the Positive and Negative Syndrome Scale and Social and Occupational Functioning Scale will be administered by a trained researcher from the study team. Non-patient participants will furnish data on mental wellbeing (Short Warwick–Edinburgh Mental Well-Being Scale), community integration (Community Integration Measure), and Social Distancing Scale. More details on each scale are reported in the following section (full measures included in Supplementary Materials).

#### 2.10.2. Questionnaire on Process of Recovery

The Questionnaire about the Process of Recovery (QPR) is a scale that measures an individual’s recovery from psychosis [25]. Items in the QPR are formed based on three main themes in the process of recovery generated based on a qualitative study conducted by Pitt et al. (2007): “rebuilding self”, “rebuilding life”, and “hope for a better future” [26]. The QPR is a self-reported questionnaire comprising 15 items scored on a five-point Likert scale: “strongly disagree”, “disagree”, “neither agree nor disagree”, “agree”, and “strongly agree”, and ranging from 0 to 4 points, respectively. The QPR was found to have excellent internal consistency when administered to persons with schizophrenia in Singapore with α = 0.934 [27].

#### 2.10.3. Short Warwick–Edinburgh Mental Well-Being Scale (SWEMWBS)

The 7-item short Warwick–Edinburgh Mental Well-Being Scale (SWEMWBS) was developed by Stewart-Brown et al. [28] based on the original Warwick–Edinburgh Mental Well-Being Scale (WEMWBS) [29], which consists of 14 items. The SWEMWBS measures mental well-being in an individual, scoring on a 5-point Likert scale: “none of the time”, “rarely”, “some of the time”, “often”, and “all of the time” from 1 to 5 points, respectively. The SWEMWBS showed high levels of internal consistency at α = 0.84 in the general population [30] and at α = 0.90 in the clinical population [31].

#### 2.10.4. Community Integration Measure (CIM)

The Community Integration Measure (CIM) measures community integration from the perspective of the individual [32]. Additionally, the CIM does not assume the relative importance of activities or relationships, unlike the Community Integration Questionnaire (CIQ); for example, the CIM scores similarly regardless of whether respondents interact with friends or family, unlike CIQ, which scores favorably toward interactions with friends. The CIM was also shown to have a high internal consistency of α = 0.87 [32].

#### 2.10.5. Social Distance Scale (SDS)

The social distance scale (SDS) assesses individuals’ responses toward mental illness [33]. The scale includes five vignettes portraying people with schizophrenia, major depressive disorder, alcohol dependence, drug dependence, and a generally troubled person. Only one vignette will be given at random to each individual being assessed. Follow-up questions will be given pertaining to the recognition of vignettes representing mental illnesses, perception of causes, perception of dangerousness, and attitudinal social distance. These questions are scored on a 4-point Likert scale; “very likely”, “somewhat likely”, “somewhat unlikely”, and “very unlikely” from 1 to 4, respectively, and “definitely” to “definitely not” from 1 to 4, respectively, for attitudinal social distance [33].

#### 2.10.6. Positive and Negative Symptom Scale (PANSS)

The positive and negative symptom scale (PANSS) measures both positive and negative symptoms of schizophrenia, referring to mental symptoms that manifest additionally and deficits of cognitive, affective, and social functioning, respectively [34]. Items in the scale are scored on a 7-point Likert scale: absent, minimal, mild, moderate, moderate-severe, severe, and extreme, from “1” to “7”, respectively. Additionally, items in the PANSS are categorized into three subscales: positive scale, negative scale, and the general psychopathology scale. Information is mainly collected via reports from caregivers and clinical interviews; the 30 items in the scale are then rated accordingly. Kay et al. also found that the PANSS scored highly in internal consistency of α = 0.73, 0.83, and 0.79 for the positive, negative, and general psychopathology scale, respectively [34].

#### 2.10.7. Social and Occupational Functioning Assessment Scale (SOFAS)

The social and occupational functioning assessment scale (SOFAS) serves to measure social and occupational functioning of an individual [35]. Additionally, the SOFAS does not assess any forms of symptoms in the person. Items in the SOFAS are rated on a 100-point scale; with every 10-point interval differing significantly in description; higher scores in SOFAS relate to better functioning and lower scores relate to worse functioning in either the social or occupational domain [35].

#### 2.10.8. Patients

Patient participants will go through two structured interviews to collect pre-workshops and post-workshops, within two weeks prior to and after the workshop series, respectively. In both interviews, measurements will be administered via a trained study team member. Additionally, data pertaining to occupied bed days and hospital admissions will be collected via administrative backend 18 months prior to and after the workshop series as part of the study’s secondary outcomes.

#### 2.10.9. Non-Patients

Non-patient participants will be given an online QuestionPro link instead of going through structured interviews. However, only measurements pertaining to mental well-being (SWEMWBS), social inclusion (CIM), and the social distance scale (SDS) will be included in the online questionnaire for the participants to self-administer. The QPR, PANSS, and SOFAS will not be administered to the non-patient participants, as the nature of the measurements will not be applicable to them.

#### 2.10.10. Sample Size Calculation

G*Power software [36] is used to estimate the sample size required for a suggested power of 95% [37]. This study will be recruiting at least 60 patient participants for the evaluation. This sample size is based on the effect sizes obtained by Meddings et al. [19]. Additionally, a 50% attrition rate is taken into consideration [38]. For non-patient participants, an effect size of 0.5 is calculated from Wilson et al. [39]. A sample size of 68 is, thus, derived for non-patient participants for this study. Unlike the patient participants, only a 20% attrition rate is factored into this calculation; a lower attrition rate is expected as the non-patient participants will only be required to complete a self-administered survey; thus, they are expected to be more willing to participate.

#### 2.10.11. Data Analysis

The quantitative data will be analyzed to compare the outcomes of interest in the participants before and after attending the Broken Crayons series of workshops. This will be achieved using the paired sample *t*-test and will be conducted with the Statistical Package for the Social Sciences (SPSS) 23 [40]. The two-tailed Wilcoxon signed-rank test will be used as an alternative if the data violate normality.

The qualitative data will be analyzed to better understand the subjective perception of workshop participants. A total of 12 to 16 participants will be recruited over three focus group discussions, or until data saturation, whichever comes first. Participants will be recruited at the end of every run of the workshop series. The audio-recorded data will be transcribed before proceeding with analysis. Thematic analysis will be used for analyzing any emerging themes regarding co-production and the experiences of the participants toward the workshop [41]. Available data of participants who choose to withdraw from the research study will still be used in the data analysis to ensure complete and comprehensive evaluation of the study. Supplementary File S2 summarizes the interview guide for the focus group discussions.

## 3. Discussion

In sum, this paper details the protocol of a project to implement and evaluate a co-produced workshop series named *Broken Crayons*. A framework for co-production is developed by the study team with a group of volunteers as a guide to operationalize co-production in the context of an early intervention program for first-episode psychosis in Singapore. Staying true to the spirit of co-production, both patient and non-patient participants will be asked to join the workshop series. As such, our evaluation protocol includes data collection from participants of these two demographics. Finally, focus group discussions will be conducted to elicit the subjective experiences of participants to understand their perspectives on attending *Broken Crayons*.

In Singapore, co-production is gaining traction in the design and delivery of mental health services in the hospital and community settings [8]. While co-production is easy to grasp conceptually, its operationalization remains challenging because of its ideals. As pragmatism and meritocracy are values highly regarded in our nation [42], endeavors that are time-consuming and not the most cost-effective are less likely to be funded. Our English counterparts have argued for the business case for co-production [43], which may be helpful in our context because the co-production can be cost-saving due to the involvement of volunteers in the community. Yet, the value of co-production not only lies in its cost-effectiveness; it is a moral imperative to create inclusive and equitable mental health interventions for persons in recovery. Co-production gives agency back to the beneficiaries of mental health interventions to make decisions; it levels the power differential between service providers and service users in mental healthcare. In recent years, there have been increasing awareness and social activism around the topic of mental health in Singapore [44]. Anchoring the call for co-production as a moral imperative for the inclusion of persons with mental health issues in their own care and for its cost-effectiveness may be a good strategy forward.

Bourne et al. [45] reported that Recovery Colleges have positive benefits for service users through using mental health services less, spending fewer days in hospital, having fewer admissions, and fewer of these admissions being compulsory. This paper equated these benefits to a cost saving of about GBP 1200 per registered student. The cost-savings projected by Bourne et al. [45] of GBP 1200 is based on non-cashable savings on the reduction of community service use and occupied bed days of the workshop participants. The savings per participant was calculated based on the total cost of service use before attending recovery college and the total cost of service use after registration for recovery college courses per participant. They found that participants who completed recovery courses had more significant cost-savings compared to those who registered but did not complete 70% of workshops.

As co-production in mental health could be used as an approach in different contexts, having a framework that is context-specific could go a long way to operationalize this approach. The framework created in this project is for posterity, a guideline to develop co-production workshops in the future so that there will not be a need to start from scratch. Future volunteers and professionals interested in co-production could use this framework as a guide. It also serves as a self-evaluation tool, where professionals can ask themselves the degree to which they are involving patients in their interventions. The various levels of involvement serve as an evaluative tool for self-reflection and improvement. It is also a “living” document, where changes and customization can be done on it to fine-tune and cater to specific co-production projects.

## 4. Conclusions

Taken together, co-production in mental health is a fairly new approach to service design and delivery in Singapore. Despite its relative novelty, we argue that co-production is more than a passing trend, but a moral imperative. Many studies have reported the social and economic consequences of living with the impact of psychoses for young people with first-episode psychosis. By collaborating with the beneficiaries of early intervention programs to design and delivery services, one could deliver more relevant and humane mental health interventions that are morally and financially sound. The implication of this project is that it contributes to the small but promising literature on the effectiveness of co-production in mental healthcare. In Singapore, this study represents the first of its kind to be conducted. This project is an effort to build the foundation of the evidence base for co-production in mental healthcare in Singapore. It is time to return the patients’ voice to its rightful place and create opportunities for them to make key decisions in their recovery journeys.

## Figures and Tables

**Table 1:** A table to summarize the gist of every workshop in the *Broken Crayons* workshop series.

Session	Activities
1	**Workshop 1: Understanding Psychosis** A psychiatrist will be giving a talk on the biological aspects of psychosis for the purpose of psychoeducation. A person in recovery will be sharing his or her recovery story toward the end of the session too. An opportunity for Q&A will be available to participants.
2 and 3	**Workshops 2 and 3: Journeying with Psychosis 1 and 2** Various everyday aspects of living with a psychotic illness (issues of medications, dealing with negative mindsets, and understanding a mind in a psychotic episode) will be covered in these workshops. More persons with a lived experience of psychosis will be invited to share their stories of recovery with the participants in creative and interactive ways.
4	**Workshop 4: What keeps you going?** This workshop will address negative symptoms of psychotic disorders to help participants find internal motivations to keep going while living with psychosis.
5	**Workshop 5: Disclosure of Medical History** This workshop will happen in the form of a panel discussion, where employers, caregivers, persons in recovery, and a moderator will come together and have a structured discussion about disclosure of medical history in mental health issues.
6	**Workshop 6: Family & Friends** This final session is a wrap-up of the workshop series, where participants will learn more about practicing gratitude and could show gratitude toward their supporters in their journeys.

**Table 2:** A self-assessment exercise to ensure meaningful, non-tokenistic involvement of peers in the development and implementation of *Broken Crayons*.

S/N	Self-Assessment Questions for Meaningful Peer Involvement (Jones, 2016) [24]	Self-Assessment
1	Have attempts been made to include peers as early as possible in planning a new initiative or program?	Yes. Peers were involved right from the beginning of the creation of broken crayons workshops.
2	Do peers have the power to make decisions and shape the programs, or are they limited to “advisory” roles?	Yes. Peers were given the power to make executive decisions about broken crayons, a democratic approach is usually taken to resolve differences.
3	Are peers financially compensated in a manner equal to non-peers?	Yes. We hired a research executive who has a lived experience to helm the program. She was compensated based on her educational qualifications and work experience. Her salary and job grade were determined using industry standards by our HR department.
4	Is there critical mass (or sufficient number) of peers involved to make a difference?	We have a thriving group of peers and caregivers volunteering in our midst to design and deliver broken crayons. Mental health professionals are in minority numbers in our volunteer group.
5	Have steps been taken to ensure that peer wellness is prioritized?	Yes, psychological safety and alliance was built between peers and peer staff to ensure that peers are well supported.
6	Has the program or organization invested in peer capacity building—e.g., paying peers to attend conferences and workshops and to learn new skills?	Yes, peer capacity building is a priority, where peers were given volunteers training and informal qualitative feedback after every workshop for continuous learning and development.
7	Have program leaders or administrators taken explicit steps to ensure that peer perspectives are valued, and that resistance to peer involvement is systematically addressed?	Program leaders have made it clear that peer involvement is the mainstay of the program and encouraged peer involvement in meaningful ways.

**Table 3:** A table to summarize five key areas for the on-boarding of volunteers.

Step	Activities		
**1**	**Volunteer induction**		
	**Low involvement**	**Medium involvement**	**High involvement**
	Go through a checklist of do’s and don’ts with the volunteers	Group 2 newcomers with 1 more experienced volunteer to learn the ropes (1:3 or 1:4 ratio) Mentor/mentee	Tag each newcomer with a more experienced volunteer to learn the ropes Welcome people in different stages of recovery as they are
**2**	**Volunteer roles**		
	**Low involvement**	**Medium involvement**	**High involvement**
	Assign fixed roles to volunteers E.g., Timekeeper, graphic design (posters), scribers	Discuss potential roles with volunteers E.g., Facilitator, curriculum developer	Share about the program Ask volunteers to contribute based on their strengths and interests Working with volunteers in whatever stage of recovery they are at
**3**	**Stage-by-stage involvement**		
	**Low involvement**	**Medium involvement**	**High involvement**
	Sitting in a session Experience a co-produced workshop or planning discussion Help with scribing	Facilitate a small segment of the workshop Icebreaker Small group facilitation Involve in a bit of planning	Take the lead in session Plan Be main facilitator Full on practical
**4**	**Development and retention**		
	**Low involvement**	**Medium involvement**	**High involvement**
	Little or no development of volunteers E.g., group debrief	Some development or training of volunteers E.g., Role-playing and “comparing notes” within the facilitators during dry run, newer facilitators being tagged to EPIP team members (e.g., a more experienced facilitator is tagged with a less experienced facilitator in a breakout room)	Long-term development plans and training and development of volunteers based on their strengths and skills
**5**	**Commitment level**		
	**Low involvement**	**Medium involvement**	**High involvement**
	No flexibility E.g., Timings are catered to organizer’s schedule and preferences.	Some flexibility E.g., offering suggestions to volunteers for session timings (e.g., give two dates), discuss possible timings together.	Catered to the volunteers’ schedule and commitments E.g., Having a contingency plan for volunteers should they have other work or last-minute commitments to attend to and cannot be there for the workshop.

## Data Availability

Not applicable.

## Supplementary Materials

The following supporting information can be downloaded at: https://www.mdpi.com/article/10.3390/ijerph192315464/s1, Supplementary file consists of scales used for the evaluation and more information on the co-production framework.
