# Supplementary material for: A Mixed-Methods Outcomes Evaluation Protocol for a Co-Produced Psychoeducation Workshop Series on Recovery from Psychosis
Source: Int J Environ Res Public Health. 2022 Nov 22;19(23):15464. doi: 10.3390/ijerph192315464 (PMC9736781; doi:10.3390/ijerph192315464)
Supplement: Supplementary file 1 [file ijerph-19-15464-s001.zip › ijerph-1918241-supplementary.pdf]

Background Information

Thank you very much for taking the time to participate in this study. Below is a simple questionnaire that will help us appreciate our respondents' profile. Please be assured that this information will be kept strictly confidential, and you will never be identified by name. This background information will help us during the analysis.

1. Please tick against your gender:

☐ Female ☐ Male

2. What age were you on your last birthday?

☐

3. What is your ethnicity?

☐ Chinese
☐ Malay
☐ Indian
☐ Eurasian
☐ Others (specify) ____________________

4. Are you currently

☐ Single / Never married
☐ Married
☐ Divorced / separated
☐ Widowed

5. What is your highest educational qualification?

☐ No formal education
☐ PSLE
☐ 'O' / 'N' level
☐ 'A' level / Pre-U
☐ Vocational Certificate
☐ Diploma
☐ Degree
☐ Post graduate degree (e.g. Masters/ PhD)
☐ Others (specify) ____________

6. What is your occupation?

- ☐ Student / Homemaker
- ☐ Unemployment
- ☐ Employed (specify) _______________
- ☐ Others (specify) _______________

7. Can you tell me your diagnosis?

- ☐ Brief Psychotic Disorder
- ☐ Schizophrenia
- ☐ Schizoaffective disorder
- ☐ Schizophreniform
- ☐ Delusional disorder
- ☐ Depression with psychotic features
- ☐ Bipolar disorder with psychotic features
- ☐ Others (specify) _____________________
- ☐ I don't know / I don't want to share

8. When were you first in contact with EPIP? (dd/mm/yy, or mm/yy or yy if you do not remember the exact date)

9. Do you have any long-term illness, health problems or disability which limits your daily activities or the work you can do? Include problems which are due to old age.

Yes	
No	

QPR

Please take a moment to consider and sum up how things stand for you at the present time, in particular the last 7 days, with regards to your mental health and recovery. Using the scale below, please circle the appropriate number that represents your degree of agreement to the statements.

1	2	3	4	5
Strongly disagree	Disagree	Neither agree nor disagree	Agree	Strongly agree

S/N	Statement					
1	I feel better about myself.	1	2	3	4	5
2	I feel able to take chances in life.	1	2	3	4	5
3	I am able to develop positive relationships with other people.	1	2	3	4	5
4	I feel part of society rather than isolated.	1	2	3	4	5
5	I am able to assert myself.	1	2	3	4	5
6	I feel that my life has a purpose.	1	2	3	4	5
7	My experienced have changes me for the better.	1	2	3	4	5
8	I have been able to come to terms with things that have happened to me in the past and move on with my life.	1	2	3	4	5
9	I am basically strongly motivated to get better.	1	2	3	4	5
10	I can recognize the positive things I have done.	1	2	3	4	5
11	I am able to understand myself better.	1	2	3	4	5
12	I can take charge of my life.	1	2	3	4	5
13	I can actively engage with life.	1	2	3	4	5
14	I can take control of aspects of my life.	1	2	3	4	5
15	I can find the time to do the things I enjoy.	1	2	3	4	5

(S)WEMWBS

Below are some statements about feelings and thoughts.

Please select the answer that best describes your experience of each over the last 2 weeks.

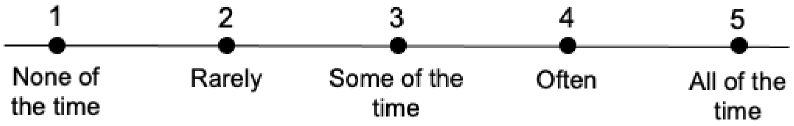

**S/N**	**Statements**					
1	I’ve been feeling optimistic about the future.	1	2	3	4	5
2	I’ve been feeling useful.	1	2	3	4	5
3	I’ve been feeling relaxed.	1	2	3	4	5
4	I’ve been dealing with problems well.	1	2	3	4	5
5	I’ve been thinking clearly.	1	2	3	4	5
6	I’ve been feeling close to other people.	1	2	3	4	5
7	I’ve been able to make up my own mind about things.	1	2	3	4	5

CIM

Please read the following statements and rate if you agree or disagree with them. “Community” refers to the group of people with whom you have most contact with. It could be at the workplace, school, family, interest groups, or religion/faith groups etc. Contact can be face-to-face or virtual. Please provide an answer that is closest to how you feel.

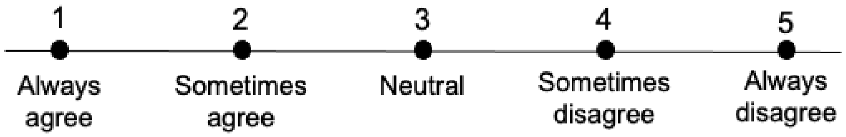

**S/N**	**Statements**					
1	I feel like part of this community, like I belong here.	1	2	3	4	5
2	I know my way around this community.	1	2	3	4	5
3	I know the rules in this community and I can fit in with them.	1	2	3	4	5
4	I feel that I am accepted in this community.	1	2	3	4	5
5	I can be independent in this community.	1	2	3	4	5
6	I like where I’m living now.	1	2	3	4	5
7	There are people I feel close to in this community.	1	2	3	4	5
8	I know a number of people in this community well enough to say hello and have them say hello back.	1	2	3	4	5
9	There are things that I can do in this community for fun in my free time.	1	2	3	4	5
10	I have something to do in this community during that main part of my day that is useful and productive.	1	2	3	4	5

LSDS

Instructions: Please read the scenarios below and answer the questions pertaining to each scenario.

A. During the last month John has started to drink more than his usual amount of alcohol. In fact, he has noticed that he needs to drink twice as much as he used to get the same effect. Several times, he has tried to cut down, or stop drinking, but he can't. Each time he has tried to cut down, he became very agitated, sweaty and he couldn't sleep, so he took another drink. His family has complained that he is often hungover, and has become unreliable-making plans one day, and cancelling them the next.

1. How likely it is that John was experiencing alcohol dependence?

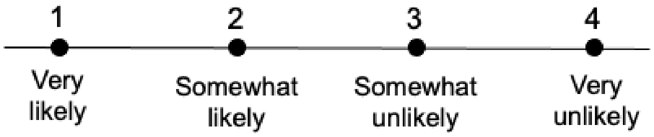

2. In your opinion, how likely is it that John's situation might be caused by:

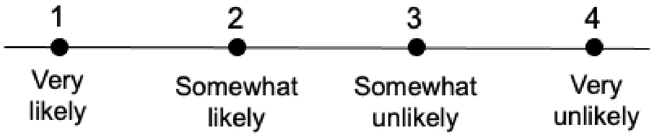

a.	The person's own bad character.	1	2	3	4
b.	A chemical imbalance in the brain	1	2	3	4
c.	The way the person was raised	1	2	3	4
d.	Stressful circumstances in the person's life	1	2	3	4
e.	A genetic or inherited problem	1	2	3	4
f.	God's will	1	2	3	4

3. In your opinion, how likely is it that John would do something violent toward other people?

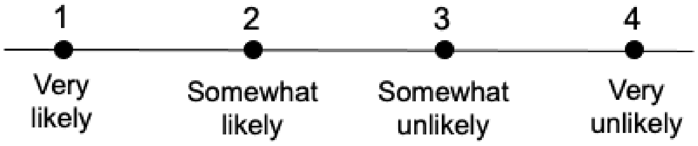

4. How willing are you to:

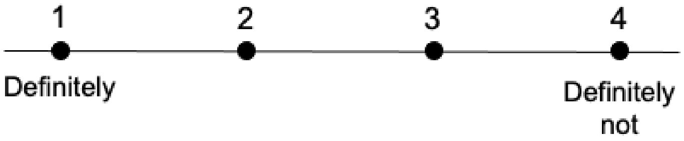

a.	Move next door to the person in the scenario?	1	2	3	4
b.	Spend an evening socialising with the person	1	2	3	4
c.	Make friends with the person	1	2	3	4
d.	Start working closely with the person	1	2	3	4
e.	Have the person marry into the family	1	2	3	4

B. For the past two weeks John has been feeling really down. He wakes up in the morning with a flat heavy feeling that sticks with him all day long. He isn't enjoying things the way he normally would. In fact, nothing gives him pleasure. Even when good things happen, they don't seem to make John happy. He pushes on through his days, but it is really hard. The smallest tasks are difficult to accomplish. He finds it hard to concentrate on anything. He feels out of energy and out of steam. And even though John feels tired, when night comes, he can't go to sleep. John feels pretty worthless and very discouraged. John's family has noticed that he hasn't been himself for about the last month and that he has pulled away from them. John just doesn't feel like talking.

1. How likely it is that John was experiencing major depression?

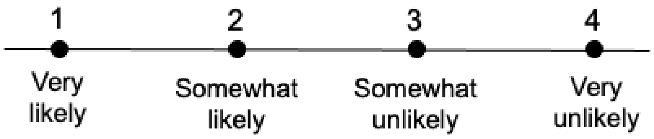

2. In your opinion, how likely is it that John's situation might be caused by:

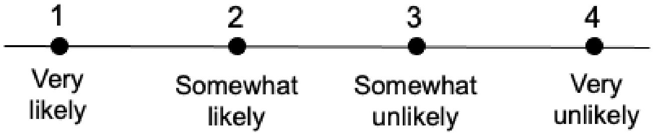

a.	The person's own bad character.	1	2	3	4
b.	A chemical imbalance in the brain	1	2	3	4

c.	The way the person was raised	1	2	3	4
d.	Stressful circumstances in the person’s life	1	2	3	4
e.	A genetic or inherited problem	1	2	3	4
f.	God’s will	1	2	3	4

3. In your opinion, how likely is it that John would do something violent toward other people?

1	2	3	4
Very likely	Somewhat likely	Somewhat unlikely	Very unlikely

4. How willing are you to:

1	2	3	4
Definitely			Definitely not

a.	Move next door to the person in the scenario?	1	2	3	4
b.	Spend an evening socialising with the person	1	2	3	4
c.	Make friends with the person	1	2	3	4
d.	Start working closely with the person	1	2	3	4
e.	Have the person marry into the family	1	2	3	4

C. Up until a year ago, life was pretty okay for John. But then, things started to change. He thought that people around him were making disapproving comments and talking behind his back. John was convinced that people were spying on him and that they could hear what he was thinking. John lost his drive to participate in his usual work and family activities and retreated to his home, eventually spending most of his day in his room. John was hearing voices even though no one else was around. These voices told him what to do and what to think. He has been living this way for six months.

1. How likely it is that John was experiencing schizophrenia?

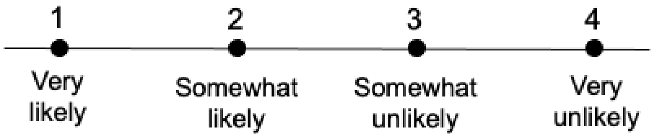

2. In your opinion, how likely is it that John's situation might be caused by:

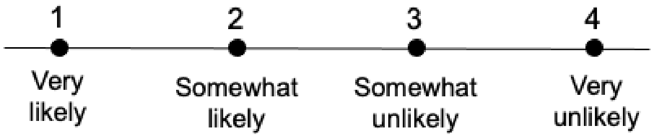

a.	The person's own bad character.	1	2	3	4
b.	A chemical imbalance in the brain	1	2	3	4
c.	The way the person was raised	1	2	3	4
d.	Stressful circumstances in the person's life	1	2	3	4
e.	A genetic or inherited problem	1	2	3	4
f.	God's will	1	2	3	4

3. In your opinion, how likely is it that John would do something violent toward other people?

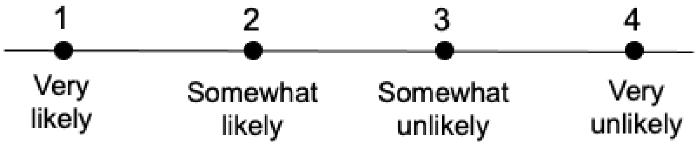

4. How willing are you to:

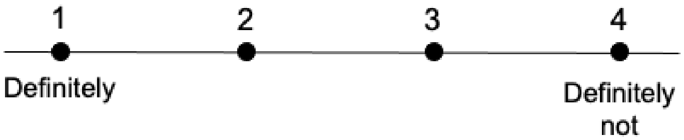

a.	Move next door to the person in the scenario?	1	2	3	4
b.	Spend an evening socialising with the person	1	2	3	4
c.	Make friends with the person	1	2	3	4

d.	Start working closely with the person	1	2	3	4
e.	Have the person marry into the family	1	2	3	4

D. A year ago, John sniffed cocaine for the first time with friends at a party. During the last few months, he has been snorting it in binges that last several days at a time. He has lost weight and often experiences chills when binging. John has spent his savings to buy cocaine. When John's friends try to talk about the changes they see, he becomes angry and storms out. Friends and family have also noticed missing possessions and suspect John has stolen them. He has tried to stop snorting cocaine, but he can't. Each time he tries to stop he feels very tired and depressed and is unable to sleep. He lost his job a month ago after not showing up for work.

1. How likely it is that John was experiencing a drug problem?

1	2	3	4
Very likely	Somewhat likely	Somewhat unlikely	Very unlikely

2. In your opinion, how likely is it that John's situation might be caused by:

1	2	3	4
Very likely	Somewhat likely	Somewhat unlikely	Very unlikely

a.	The person's own bad character.	1	2	3	4
b.	A chemical imbalance in the brain	1	2	3	4
c.	The way the person was raised	1	2	3	4
d.	Stressful circumstances in the person's life	1	2	3	4
e.	A genetic or inherited problem	1	2	3	4
f.	God's will	1	2	3	4

3. In your opinion, how likely is it that John would do something violent toward other people?

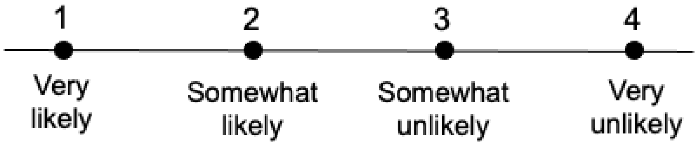

4. How willing are you to:

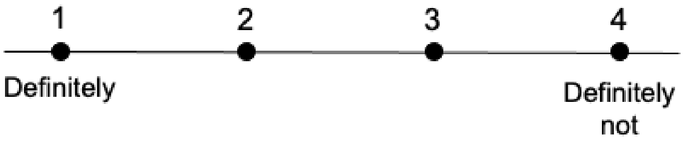

a.	Move next door to the person in the scenario?	1	2	3	4
b.	Spend an evening socialising with the person	1	2	3	4
c.	Make friends with the person	1	2	3	4
d.	Start working closely with the person	1	2	3	4
e.	Have the person marry into the family	1	2	3	4

E. Up until a year ago, life was pretty okay for John. While nothing much was going wrong in John's life, he sometimes feels worried, a little sad, or has trouble sleeping at night. John feels that at times things bother him more than they bother other people and that when things go wrong, he sometimes gets nervous or annoyed. Otherwise, John is getting along pretty well. He enjoys being with other people and although John sometimes argues with his family, John has been getting along pretty well with his family.

1. In your opinion, how likely is it that John’s situation might be caused by:

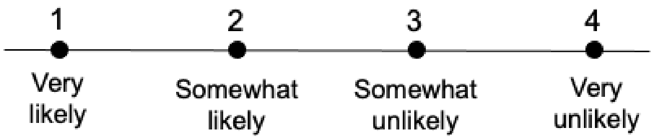

a.	The person’s own bad character.	1	2	3	4
b.	A chemical imbalance in the brain	1	2	3	4
c.	The way the person was raised	1	2	3	4
d.	Stressful circumstances in the person’s life	1	2	3	4
e.	A genetic or inherited problem	1	2	3	4

f.	God’s will	1	2	3	4

2. In your opinion, how likely is it that John would do something violent toward other people?

1	2	3	4
Very likely	Somewhat likely	Somewhat unlikely	Very unlikely

3. How willing are you to:

1	2	3	4
Definitely			Definitely not

a.	Move next door to the person in the scenario	1	2	3	4
b.	Spend an evening socialising with the person	1	2	3	4
c.	Make friends with the person	1	2	3	4
d.	Start working closely with the person	1	2	3	4
e.	Have the person marry into the family	1	2	3	4
